# Incidence and management of metabolic acidosis with sodium bicarbonate in the ICU: An international observational study

**DOI:** 10.1186/s13054-020-03431-2

**Published:** 2021-02-02

**Authors:** Tomoko Fujii, Andrew A. Udy, Alistair Nichol, Rinaldo Bellomo, Adam M. Deane, Khaled El-Khawas, Naorungroj Thummaporn, Ary Serpa Neto, Hannah Bergin, Robert Short-Burchell, Chin-Ming Chen, Kuang-Hua Cheng, Kuo-Chen Cheng, Clemente Chia, Feng-Fan Chiang, Nai-Kuan Chou, Timothy Fazio, Pin-Kuei Fu, Victor Ge, Yoshiro Hayashi, Jennifer Holmes, Ting-Yu Hu, Shih-Feng Huang, Naoya Iguchi, Sarah L. Jones, Toshiyuki Karumai, Shinshu Katayama, Shih-Chi Ku, Chao-Lun Lai, Bor-Jen Lee, Wen-Jinn Liaw, Chelsea T. W. Ong, Lisa Paxton, Chloe Peppin, Owen Roodenburg, Shinjiro Saito, John D. Santamaria, Yahya Shehabi, Aiko Tanaka, Ravindranath Tiruvoipati, Hsiao-En Tsai, An-Yi Wang, Chen-Yu Wang, Yu-Chang Yeh, Chong-Jen Yu, Kuo-Ching Yuan, for the SODA-BIC investigators, Tomoko Fujii, Andrew A. Udy, Adam M. Deane, Alistair Nichol, Rinaldo Bellomo, Ary Serpa Neto, Khaled El-Khawas, Naorungroj Thummaporn, Lisa Paxton, Timothy Fazio, Robert Short-Burchell, Allison Bone, Hannah Bergin, Sarah Jones, Jennifer Holmes, John Santamaria, Chloe Peppin, Yahya Shehabi, Ravindranath Tiruvoipati, Victor Ge, Lee-Anne Clavarino, Chelsea Ong, Owen Roodenburg, Steven Hirth, Aiko Tanaka, Naoya Iguchi, Shinshu Katayama, Jun Shima, Fumie Takatsudo, Kumie Suzuki, Shinjiro Saito, Toshiyuki Karumai, Yoshiro Hayashi, Yu-Chang Yeh, Chong-Jen Yu, Shih-Chi Ku, Nai-Kuan Chou, Ting-Yu Hu, Kuang-Hua Cheng, Chao-Lun Lai, Hsiao-En Tsai, Kuo-Ching Yuan, An-Yi Wang, Shih-Feng Huang, Wen-Jinn Liaw, Kuo-Chen Cheng, Chin-Ming Chen, Bor-Jen Lee

**Affiliations:** 1 Department of Epidemiology and Preventive Medicine, Australian and New Zealand Intensive Care Research Centre, Monash University, 553 St Kilda Rd, Melbourne, VIC 3004 Australia; 2 Intensive Care Unit, Jikei University Hospital, Tokyo, Japan; 3 Department of Intensive Care and Hyperbaric Medicine, The Alfred, Melbourne, VIC Australia; 4 School of Medicine and Medical Sciences, University College Dublin, Dublin, Ireland; 5 Department of Intensive Care, Austin Hospital, Heidelberg, VIC Australia; 6 Centre for Integrated Critical Care, Melbourne Medical School, University of Melbourne, Melbourne, VIC Australia; 7 Melbourne Medical School, Department of Medicine, The University of Melbourne, Royal Melbourne Hospital, Parkville, VIC Australia; 8 Department of Critical Care, Siriraj Hospital, Mahidol University, Bangkok, Thailand; 9 Department of Critical Care Medicine, Hospital Israelita Albert Einstein, São Paulo, Brazil; 10 Intensive Care Unit, Royal Darwin Hospital, Darwin, NT Australia; 11 Intensive Care Unit, University Hospital Geelong, Barwon Health, Geelong, VIC Australia; 12 Department of Intensive Care Medicine, Chi-Mei Medical Center, Tainan, Taiwan; 13 Department of Critical Care Medicine, Mackay Memorial Hospital Taipei Branch, Taipei, Taiwan; 14 Division of Internal & Critical Care Medicine, Taichung Veterans General Hospital, Taichung, Taiwan; 15 Department of Surgery, National Taiwan University Hospital, Taipei, Taiwan; 16 Health Intelligence, Royal Melbourne Hospital, Parkville, VIC Australia; 17 Intensive Care Unit, Peninsula Health, Frankston, VIC Australia; 18 Department of Intensive Care Medicine, Kameda Medical Center, Chiba, Japan; 19 Intensive Care Unit, St Vincent’s Hospital Melbourne, Fitzroy, VIC Australia; 20 Chung-Shan Medical University Hospital, Taichung, Taiwan; 21 Department of Anesthesiology and Intensive Care Medicine, Graduate School of Medicine, Osaka University, Osaka, Japan; 22 Department of Anesthesiology and Intensive Care Medicine, Jichi Medical University School of Medicine, Tochigi, Japan; 23 Division of Pulmonary and Critical Care Medicine, Department of Internal Medicine, National Taiwan University Hospital, Taipei, Taiwan; 24 Department of Internal Medicine, National Taiwan University Hospital Hsin-Chu Branch, Hsin-Chu, Taiwan; 25 Intensive Care Services, Eastern Health, Box Hill, VIC, Australia; 26 Critical Care and Perioperative Services, Monash Health, Melbourne, VIC Australia; 27 Critical Care Research, Monash Health School of Clinical Sciences, Monash University, Clayton, VIC Australia; 28 Department of Critical Care Medicine, Taipei Medical University Hospital, Taipei, Taiwan; 29 Department of Emergency Medicine, School of Medicine, College of Medicine, Taipei Medical University, Taipei, Taiwan; 30 Department of Anesthesiology, National Taiwan University Hospital, Taipei, Taiwan; 31 Department of Internal Medicine, National Taiwan University Hospital, Taipei, Taiwan; 32 Department of Internal Medicine, Park Branch, National Taiwan University Hospital Biomedical, Hsin-Chu, Taiwan

**Keywords:** Metabolic acidosis, Sodium bicarbonate, Intensive care unit, Vasopressor, Mortality, Observational study

## Abstract

**Background:**

Metabolic acidosis is a major complication of critical illness. However, its current epidemiology and its treatment with sodium bicarbonate given to correct metabolic acidosis in the ICU are poorly understood.

**Method:**

This was an international retrospective observational study in 18 ICUs in Australia, Japan, and Taiwan. Adult patients were consecutively screened, and those with early metabolic acidosis (pH < 7.3 and a Base Excess < –4 mEq/L, within 24-h of ICU admission) were included. Screening continued until 10 patients who received and 10 patients who did not receive sodium bicarbonate in the first 24 h (early bicarbonate therapy) were included at each site. The primary outcome was ICU mortality, and the association between sodium bicarbonate and the clinical outcomes were assessed using regression analysis with generalized linear mixed model.

**Results:**

We screened 9437 patients. Of these, 1292 had early metabolic acidosis (14.0%). Early sodium bicarbonate was given to 18.0% (233/1292) of these patients. Dosing, physiological, and clinical outcome data were assessed in 360 patients. The median dose of sodium bicarbonate in the first 24 h was 110 mmol, which was not correlated with bodyweight or the severity of metabolic acidosis. Patients who received early sodium bicarbonate had higher APACHE III scores, lower pH, lower base excess, lower PaCO_2_, and a higher lactate and received higher doses of vasopressors. After adjusting for confounders, the early administration of sodium bicarbonate was associated with an adjusted odds ratio (aOR) of 0.85 (95% CI, 0.44 to 1.62) for ICU mortality. In patients with vasopressor dependency, early sodium bicarbonate was associated with higher mean arterial pressure at 6 h and an aOR of 0.52 (95% CI, 0.22 to 1.19) for ICU mortality.

**Conclusions:**

Early metabolic acidosis is common in critically ill patients. Early sodium bicarbonate is administered by clinicians to more severely ill patients but without correction for weight or acidosis severity. Bicarbonate therapy in acidotic vasopressor-dependent patients may be beneficial and warrants further investigation.

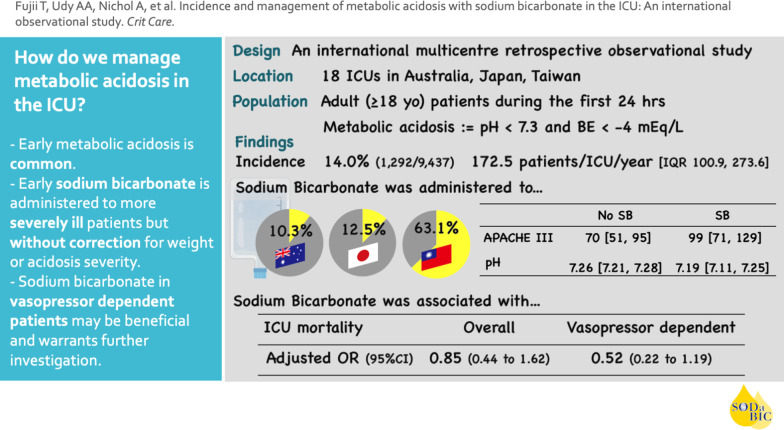

## Introduction

Metabolic acidosis is a major acid–base derangement in critically ill patients [1, 2]. Metabolic acidosis may impair cardiovascular contractility, which may contribute to reduced oxygen delivery to tissues, induce insulin resistance and may be associated with increased mortality [3, 4]. The treatment of metabolic acidosis is focused on addressing the underlying condition, i.e., shock. While the intravenous administration of sodium bicarbonate in vitro can correct acidosis, its use or effect in the clinical context is unclear. In this regard, some clinicians recommend the administration of sodium bicarbonate to patients with significant metabolic acidosis [5, 6]. These recommendations are based on the assumption that sodium bicarbonate might restore normal cardiovascular function and subsequently improve oxygen delivery [7, 8] and help correct a low pH. This effect of bicarbonate on pH is supported by a recently conducted systematic review [9]. However, in a low pH environment, hemoglobin plays a role as a buffer, by binding hydrogen ion and unloading oxygen to peripheral tissue. Administration of sodium bicarbonate may interfere with this buffering effect. Whether the intravenous administration of sodium bicarbonate to correct metabolic acidosis in vivo has beneficial effects on the cardiovascular system, i.e., vasopressor sparing effect, let alone patient-centered outcomes, i.e., mortality, remains controversial [9].

A recent multi-center, open-label, phase 3 clinical trial (*n* = 389) published in 2018 assessed the effect of sodium bicarbonate therapy in critically ill patients with severe metabolic acidosis [10]. This trial reported that targeting a pH above 7.3 with intravenous sodium bicarbonate did not significantly reduce overall 28-day mortality. However, it also reported a reduction in the use of renal replacement therapy (RRT), a secondary outcome, with such treatment. These findings have renewed interest in the role of bicarbonate therapy in patients with metabolic acidosis. However, it is unclear whether these findings are relevant and applicable to clinicians and patients in other clinical settings, because the current management of metabolic acidosis in critically ill patients is unknown.

Accordingly, we aimed to investigate the incidence and current clinical management of metabolic acidosis and the use of intravenous sodium bicarbonate therapy in the ICU in a multicenter, international setting and assess the association of such therapy with relevant biochemical and clinical outcomes.

## Methods

This was an international retrospective observational study conducted in ICUs from Australia, Japan, and Taiwan. Ethics approval was obtained from the institutional ethics committees for each participating study site.

We included adults aged 18 years or older who manifested metabolic acidosis within 24 h of ICU admission (early metabolic acidosis). Metabolic acidosis was defined as the simultaneous presence of pH < 7.3 and base excess (BE) < -4 mmol/L on arterial blood gas analysis. The time zero of the study was defined when metabolic acidosis was first diagnosed in the ICU. We screened patients who were admitted to each study ICU from Nov 11^th^ in 2017 through Dec 2^nd^, 2019, consecutively for eligibility. The screening continued until these patients could be identified: 10 patients who received sodium bicarbonate within 24 h of metabolic acidosis (the SB group) and 10 patients who did not receive sodium bicarbonate within the time frame (the No SB group) (Additional file 1: Suppl Fig. 1). The incidence of metabolic acidosis in a study ICU was calculated as the number of eligible patients divided by screening period (years). Granular data concerning dosing, physiological and biochemical patterns, and clinical outcomes were then collected on these patients. The primary outcome was ICU mortality. The secondary outcomes were hospital mortality, vasopressor doses at 6 h and 24 h, mean arterial pressures at 6 h and 24 h, and delta mean arterial pressure per vasopressor dose at 6 and 24 h.

As there are various concentrations of sodium bicarbonate solution used worldwide, e.g., bicarbonate-buffered Ringer’s solution containing 28 mEq/L of bicarbonate, we counted the administration of sodium bicarbonate only when a solution contained sodium bicarbonate at > 50 mmol/L, which is more than twice higher than physiological concentrations. When an eligible patient did not receive sodium bicarbonate within 24 h of metabolic acidosis (early bicarbonate therapy) but received it after 24 h, the patient was included in the control group.

Patient characteristics and admission information were retrieved from institutional databases or relevant medical records. Physiological status and interventions provided in the ICU over the first 24 h of metabolic acidosis were retrieved from ICU charts. All data were collected by staff at each study site and were entered into a secure web-based application of online databases, REDCap [11] (ver 10.0.11, Vanderbilt University, U.S.A.) hosted by Monash University (Victoria, Australia).

### Statistical analysis

Numerical data were summarized as medians with interquartile ranges, and categorical data were presented as counts and percentages. The total vasopressor dose was calculated as the sum of the amount of noradrenaline, adrenaline, and the converted dose of vasopressin using a previously published conversion scale [12]. Variables in the two treatment groups were compared using the Mann–Whitney U test or Fisher’s exact test. The trajectory of the biochemical or physiological data was plotted using box plots. The trend over time in each group was tested using linear regression analysis with a mixed model accounting for the random effects of an individual patient. The interaction between time and the intervention groups was assessed by p value for interaction similarly calculated from a linear regression analysis. A p for interaction < 0.1 was considered to be statistically significant. To assess vasopressor responsiveness to achieve hemodynamic stability, a calculated variable (mean arterial pressure divided by vasopressor dose plus 1) was used. To correct a vasopressor dose of 0 in the denominator, 1 was added to all vasopressor dose. The association between the administration of sodium bicarbonate and clinical outcomes, i.e., mortality and hemodynamic indices, were assessed using generalized linear regression analysis with a mixed model accounting for the random effect of each site. To adjust for confounding factors, age, sex, APACHE III score, and vasopressor doses at the diagnosis of metabolic acidosis were added to the model for ICU and hospital mortality. For other clinical outcomes, age, sex, APACHE III score, baseline levels of each outcome measures at the diagnosis of metabolic acidosis were added to the model. Considering the possible effect of sodium bicarbonate on hemodynamic stability, a subgroup analysis was conducted in patients who were vasopressor dependent at the diagnosis of metabolic acidosis. Also, we performed a subgroup analysis in patients with severe metabolic acidosis defined by pH ≤ 7.20, PaCO_2_ ≤ 45 mm Hg, HCO_3_ ≤ 20 mmol/L and Lactate ≥ 2 mmol/L in addition to BE < -4 mEq/L; and in patients with acute kidney injury (AKI) stage 2 or 3 to validate the findings in the previous trial [10]. Patient data after ICU discharge were not retrieved, and the missing data were not imputed as the missing occurred, not at random. A two-tailed p value < 0.05 was statistically significant otherwise stated. R version 4.0.1 (2020, R Foundation for Statistical Computing, Vienna, Austria) was used.

## Results

### Prevalence and incidence of metabolic acidosis

Screening data and patient-level data were available from 18 ICUs from the three countries. The total screening period at all sites was 2636 days, with 9437 patients assessed for eligibility. Of these, 1292 were identified as manifesting metabolic acidosis within 24 h of ICU admission (14.0%). The median incidence of metabolic acidosis at a study ICU was 172.5 patients/year (median; IQR, 100.9 to 273.6). Overall, intravenous sodium bicarbonate was given to 18.0% (233/1292) of patients with early (within 24 h) metabolic acidosis, but the proportion of bicarbonate administration varied markedly by the site (highest: 78.2%; lowest: 5.1%) and by country (Taiwan, 63.1%; Japan, 12.5%; Australia, 10.3%;)

### Patient characteristics

Each ICU provided data for the first 10 patients who received SB and data for the first 10 patients who did not receive SB (*N* = 360, in total). The characteristics of these 360 patients are presented in Table 1. Patients in the SB group were more likely to be admitted from the Emergency Department or ward and to have a higher APACHE III score. Patients who were on chronic hemodialysis were less likely to receive sodium bicarbonate. The prevalence of acute kidney injury (AKI) was similar at baseline (Table 1). Arterial blood gas analysis at the diagnosis of metabolic acidosis showed that patients in the SB group had lower pH, lower PaCO_2_, lower HCO_3_, lower BE, and higher lactate levels, resulting in a smaller strong-ion difference (SID) than patients in the No SB group (Table 2). The cardiac index was rarely monitored, and higher doses of vasopressors were administered in the SB group at the time of metabolic acidosis diagnosis (Table 3). Sixty-two percent of patients were on invasive mechanical ventilation in both groups (Table 3).

**Table 1:** Patient characteristics at ICU admission

	No SB (*N* = 180)	SB (*N* = 180)	*p* Value
Age, years	67.0 [55.6, 77.2]	65.8 [54.3, 76.2]	0.43
Sex, male	119 (66.1)	100 (55.6)	0.05
Weight, kg	67.0 [55.7, 84.0]	64.0 [53.1, 80.3]	0.12
Height, cm	165 [158, 172]	165 [157, 172]	0.74
BMI, kg/m^2^	24.0 [20.9, 29.0]	23.2 [20.5, 27.5]	0.18
ICU admission source			0.002
Operation theatre	72 (40.0)	40 (22.2)	
Emergency department	64 (35.6)	84 (46.7)	
Ward	29 (16.1)	43 (23.9)	
Other hospital	13 ( 7.2)	9 ( 5.0)	
ICU in other hospital	2 ( 1.1)	4 ( 2.2)	
Treatment goals on admission			0.26
Full active management	164 (91.1)	154 (85.6)	
Treatment limitation order	14 ( 7.8)	20 (11.1)	
Palliative care	2 ( 1.1)	5 ( 2.8)	
Potential organ donation	0 ( 0.0)	1 ( 0.6)	
Cardiac arrest in 24 h prior to ICU admission			0.19
Yes	26 (14.5)	36 (20.0)	
No	153 (85.5)	143 (79.4)	
Unknown	0 ( 0.0)	1 ( 0.6)	
Chronic hemo-dialysis/peritoneal-dialysis	23 (12.9)	8 ( 4.5)	0.01
Baseline creatinine, mmol/L	92.5 [70.0, 189.2]	97.0 [61.9, 147.7]	0.38
AKI			0.07
No AKI	57 (47.9)	39 (35.1)	
Stage 1	28 (23.5)	22 (19.8)	
Stage 2	8 ( 6.7)	11 ( 9.9)	
Stage 3	26 (21.8)	39 (35.1)	
APACHE III score	70 [51, 95]	99 [71, 129]	< 0.001

SB denotes sodium bicarbonate; BMI denotes body mass index; ICU, intensive care unit; AKI, acute kidney injury; APACHE, Acute Physiology And Chronic Health Evaluation

**Table 2:** Arterial blood gas analysis and electrolytes at the first diagnosis of metabolic acidosis

	No SB (*N* = 180)	SB (*N* = 180)	*p* Value
pH	7.26 [7.21, 7.28]	7.19 [7.11, 7.25]	< 0.001
PaO2, mmHg	110.5 [84.8, 162.5]	118.2 [84.6, 203.3]	0.25
PaO2/FiO2	277.1 [187.0, 377.9]	282.0 [148.2, 418.1]	0.86
PaCO2, mmHg	43.0 [37.5, 48.0]	38.6 [29.3, 45.4]	< 0.001
HCO3, mEq/L	18.6 [16.0, 20.9]	14.4 [10.4, 18.0]	< 0.001
Base Excess, mEq/L	− 8.0 [− 10.9, − 5.9]	− 12.6 [− 18.1, − 9.1]	< 0.001
SID, mEq/L	30.7 [27.5, 33.8]	28.0 [24.1, 32.0]	0.001
Sodium, mEq/L	137.0 [135.0, 139.3]	137.0 [134.0, 140.5]	0.98
Potassium, mEq/L	4.3 [3.8, 4.9]	4.4 [3.8, 5.0]	0.38
Chloride, mEq/L	109.0 [105.0, 112.8]	108.0 [103.0, 113.0]	0.50
Lactate, mmol/L	2.5 [1.4, 4.5]	4.4 [1.5, 9.3]	0.001
Ionized Calcium, mmol/L	1.1 [1.1, 1.2]	1.1 [1.0, 1.2]	0.73

SB denotes sodium bicarbonate; SID, strong ion difference = Sodium + Potassium + Ionized calcium–Chloride–Lactate

pH, PaO2, PaCO2, HCO3, Base Excess, and lactate were only measured for arterial blood samples

**Table 3:** ICU interventions at the time of metabolic acidosis diagnosis

	No SB (*N* = 180)	SB (*N* = 180)	*p* Value
Vasopressor dependent	77 (42.8)	110 (61.1)	0.001
Vasopressor dose among vasopressor-dependent patients, μg/kg/min	0.12 [0.05, 0.28]	0.25 [0.13, 0.47]	< 0.001
Cardiac index monitored	19 (10.6)	14 (7.8)	0.37
Cardiac index, L/min/m^2^	2.6 [2.2, 3.0]	2.6 [2.0, 3.3]	0.74
Respiratory support			0.16
Not on mechanical ventilation	63 (35.0)	59 (32.8)	
Invasive mechanical ventilation	112 (62.2)	112 (62.2)	
Noninvasive mechanical ventilation	5 (2.8)	4 (2.2)	
ECMO	0 (0.0)	5 (2.8)	
Neuromuscular blockade	26 (14.6)	18 (10.1)	0.20
Renal replacement therapy			0.54
Not on RRT	167 (93.3)	164 (91.1)	
CRRT	8 (4.5)	13 (7.2)	
IRRT	4 (2.2)	3 (1.7)	

SB denotes sodium bicarbonate; ECMO, extracorporeal membrane oxygenation; RRT, renal replacement therapy; CRRT, continuous renal replacement therapy; IRRT, intermittent renal replacement therapy

### Characteristics of early sodium bicarbonate therapy

Early (first 24 h) sodium bicarbonate was given at a median concentration of 918 mmol/L (IQR, 833 to 1000) at the median rate of 100 mmol/hr (IQR, 63 to 125), at a median time of 2.0 h (IQR, 0.4 to 5.1) after fulfilling early metabolic acidosis criteria in ICU. The median total dose of early sodium bicarbonate was 110 mmol (IQR, 100 to 208). Early sodium bicarbonate was administered by bolus (< 60 min, 65.9%) or by continuous infusion (≥ 60 min, 27.3%), or, less commonly, by bolus followed by continuous infusion. The total amount of early sodium bicarbonate did not correlate with body weight, base excess, or bicarbonate levels (Additional file 1: Suppl Fig. 2).

### Changes in acid–base, biochemical and physiological variables

**Fig. 1:**
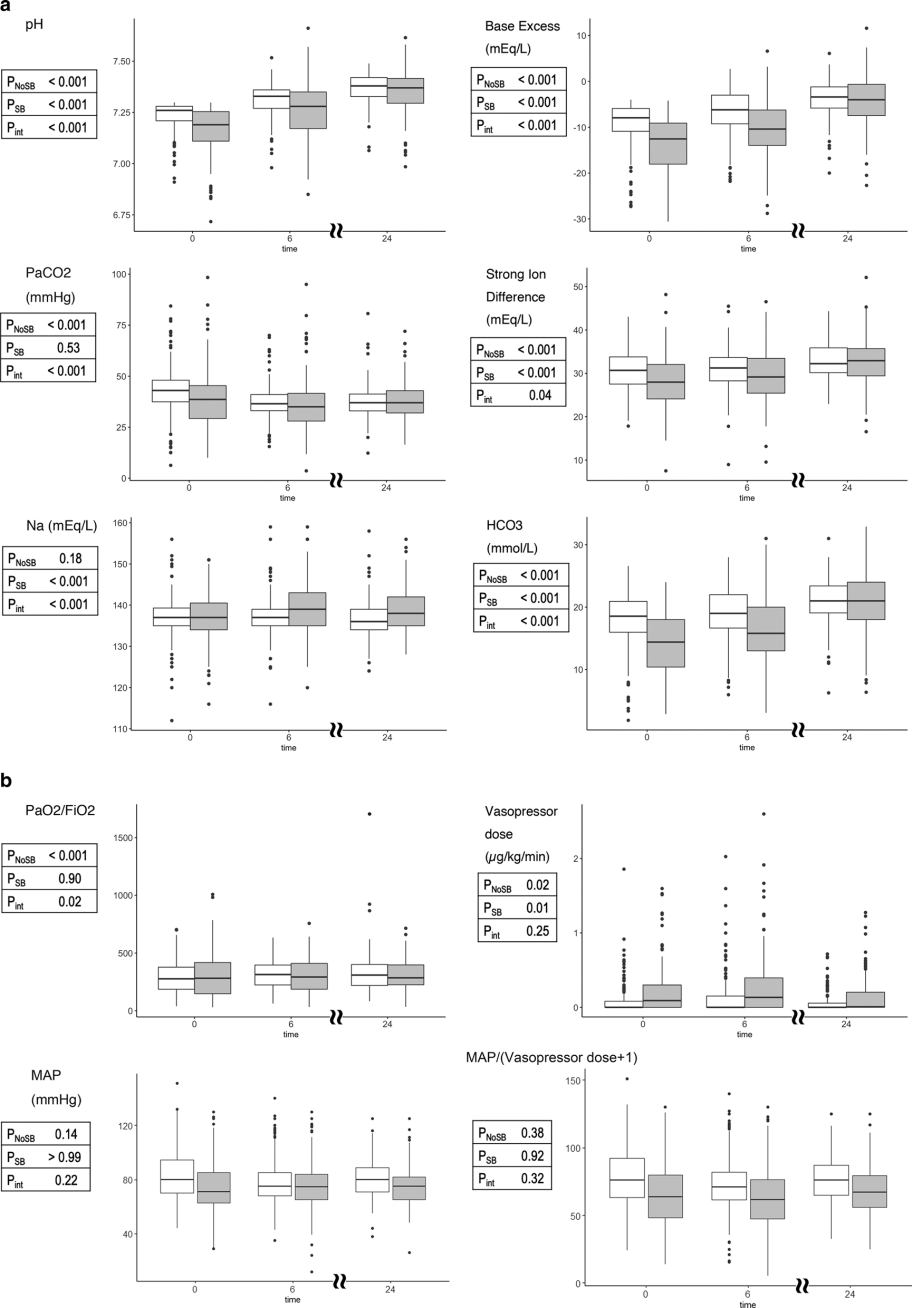
Trajectory of biochemical(**a**) or physiological(**b**) variables during the first 24 h of metabolic acidosis by treatment group. White box, No SB group. Gray box, SB group. P_NoSB_ denotes p for trend within patients who did not receive sodium bicarbonate for metabolic acidosis. P_SB_, p for trend within patients who received sodium bicarbonate. P_int_, p for interaction between treatment groups and the variables

Overall, in the early SB group, pH, BE, SID, sodium, and HCO_3_ increased, and vasopressor dose decreased over the first 24 h of metabolic acidosis (Fig. 1). Similarly, in the no SB group, pH, BE, SID, HCO_3_, and PaO_2_/FiO_2_ ratio increased, and PaCO_2_ and vasopressor dose also decreased over time (Fig. 1). However, there was an interaction between time (24 h) and treatment group for pH, BE, PaCO_2_, SID, sodium, HCO3, and PaO_2_/FiO_2_, such that all these variables increased significantly more with bicarbonate administration than without (Fig. 1). In contrast, mean arterial pressure or vasopressor doses did not behave differently according to SB administration (Fig. 1). The need for RRT increased similarly over time in both groups (Additional file 1: Suppl Fig. 3).

### Clinical outcomes

After adjusting for confounding factors and despite greater illness severity, the early administration of sodium bicarbonate was not significantly associated with ICU mortality (adjusted OR, 0.85; 95%CI, 0.44 to 1.62) or hospital mortality, vasopressor dose at 6 h or 24 h, or mean arterial pressure at 6 h or 24 h in the overall study population (Table 4).

**Table 4:** Association between administration of sodium bicarbonate and clinical outcomes

	Overall population (*N* = 360)			Vasopressor dependent at the diagnosis of metabolic acidosis (*N* = 187)			Severe metabolic acidosis at the time of diagnosis (*N* = 46)		
	aOR, β	95% CI	*p* Value	aOR, β	95% CI	*p* Value	aOR, β	95% CI	*p* Value
ICU mortality	0.85	0.44 to 1.62	0.63	0.52	0.22 to 1.19	0.13	0.86	0.08 to 9.03	0.90
Hospital mortality	0.96	0.51 to 1.76	0.89	0.84	0.38 to 1.85	0.68	1.07	0.09 to 12.3	0.96
Vasopressor dose at 6 h	–0.02	–0.08 to 0.03	0.36	0.01	–0.07 to 0.09	0.81	–0.23	–0.50 to 0.03	0.11
Vasopressor dose at 24 h	0.04	–0.01 to 0.08	0.10	0.04	–0.03 to 0.11	0.24	–0.004	–0.16 to 0.16	0.97
Mean arterial pressure at 6 h	2.85	–0.50 to 6.15	0.09	5.99	1.84 to 10.2	0.01	6.60	–3.65 to 17.2	0.23
Mean arterial pressure at 24 h	–1.07	–4.28 to 2.13	0.52	–1.03	–5.21 to 3.15	0.63	8.90	–1.44 to 19.2	0.13
Delta mean arterial pressure per vasopressor dose at 6 h	3.66	–0.78 to 8.09	0.11	8.87	3.34 to 14.48	0.002	8.53	–8.60 to 25.4	0.35
Delta mean arterial pressure per vasopressor dose at 24 h	1.26	–3.68 to 6.22	0.28	3.65	–2.56 to 9.88	0.26	13.7	0.79 to 26.6	0.06

Delta mean arterial pressure per vasopressor dose = mean arterial pressure at 24 h/(vasopressor dose at 24 h + 1) – mean arterial pressure at 6 h/(vasopressor dose at 6 h + 1)

### Subgroup analysis: Patients on vasopressors

Seventy-seven patients (42.8%) in the No SB group and 110 patients (61.1%) in the early SB group were on vasopressors at the time of the first demonstration of metabolic acidosis in ICU. Patients on vasopressors in the early SB group were younger and had a higher APACHE III score at ICU admission (Additional file 1: Suppl Table 1). These patients had lower pH, lower BE, lower SID, and higher lactate levels at the diagnosis of metabolic acidosis (Additional file 1: Suppl Table 2). They also received higher doses of vasopressors (Additional file 1: Suppl Table 3). The OR for ICU mortality among these patients was 0.52 (95%CI, 0.22 to 1.19) for those receiving sodium bicarbonate. Early sodium bicarbonate was also associated with higher mean arterial pressure and increased vasopressor responsiveness at 6 h in this subgroup (Table 4).

### Subgroup analysis: Severe metabolic acidosis

Seven patients (3.9%) in the No SB group and 39 patients (21.7%) in the early SB group had severe metabolic acidosis (Additional file 1: Suppl Fig. 4). Patients with severe metabolic acidosis in the early SB group were less likely to have chronic kidney disease (Additional file 1: Suppl Table 4). They had lower BE or bicarbonate levels and higher PaO_2_/FiO_2_ ratio at the diagnosis of metabolic acidosis (Additional file 1: Suppl Table 5), although none of them reached statistical significance. They received higher doses of vasopressors (Additional file 1: Suppl Table 6). The aOR for ICU mortality was 0.86 (95%CI, 0.08 to 9.03) in those receiving sodium bicarbonate (Table 4).

### Subgroup analysis: Stage 2 or 3 AKI

Thirty-four (18.9%) patients in the No SB group and 50 patients (27.8%) in the early SB group had stage 2 or 3 AKI (Additional file 1: Suppl Table 7). Patients with AKI in the early SB group were less likely to have chronic kidney disease but were more severely ill (Additional file 1: Suppl Table 7). They had lower pH, BE or bicarbonate levels and higher lactate levels at the time of diagnosis of metabolic acidosis (Additional file 1: Suppl Table 8). They also received higher doses of vasopressors (Additional file 1: Suppl Table 9). The aOR for ICU mortality was 0.72 (95%CI, 021 to 2.58) in those receiving sodium bicarbonate (Additional file 1: Suppl Table 10).

## Discussion

### Key findings

Early metabolic acidosis was common in our study cohort, and early intravenous sodium bicarbonate was variably used in such patients across the study ICUs. Most patients who received sodium bicarbonate did so as a bolus dose. However, the prescription was not adjusted for body weight or the severity of metabolic acidosis. Patients who received sodium bicarbonate had a higher severity of critical illness and more severe metabolic acidosis. However, their biochemical parameters and PaO2/FiO2 ratios improved more rapidly than for patients who did not receive bicarbonate. Early sodium bicarbonate was not significantly associated with improved clinical outcomes overall. However, in patients with vasopressor dependency at diagnosis, sodium bicarbonate was associated with higher mean arterial pressures within six hours and a favorable, but not statistically significant, aOR for ICU mortality.

### Relationship with prior studies

A recent systematic review reported that the biochemical and physiological effects of sodium bicarbonate had been poorly examined [9]. Such a lack of data significantly limits our understanding of how much and how fast sodium bicarbonate should effectively be given to patients with metabolic acidosis. The current study confirms that the speed of administration, the mode (bolus or infusion or both), and the dose are highly variable and not related to any clinical or biochemical parameters. Moreover, despite the suggested rationale for administering sodium bicarbonate [9], there is a paucity of recent data exploring any effect on hemodynamic indices. Thus, our study is the first contemporary work to indicate that this therapy might increase mean arterial pressure early in patients on vasopressors at the diagnosis of metabolic acidosis, a finding consistent with the putative effect on the cardiovascular system.

The effects of sodium bicarbonate on clinical outcomes have been rarely reported, except for a recent randomized clinical trial conducted in France [10]. This trial reported that administration of sodium bicarbonate for severe metabolic acidosis defined by pH ≤ 7.20, PaCO_2_ ≤ 45 mm Hg, and bicarbonate concentration ≤ 20 mmol/L decreased the need for RRT by 16.7%. Moreover, in patients with AKI, sodium bicarbonate reduced mortality by 17.7%. The results suggest promising effects of sodium bicarbonate therapy for metabolic acidosis; however, the trial was not blinded, creating the possibility of performance bias and focused only on severe metabolic acidosis. Thus, the applicability and relevance of the findings are unclear for most patients in the ICU. The current study demonstrated that mild to moderate metabolic acidosis defined by pH < 7.3 and BE < -4 mEq/L were frequently observed in the ICU, and sodium bicarbonate was commonly used in such patients.

The mortality benefit observed in the subgroup of patients with AKI in the previous trial [10] was not replicated in this observational study. However, this might be attributable to the small sample size of the subgroup analysis. Another difference from the previous trial was the amount of bicarbonate provided in the 24 h. Patients in the bicarbonate group of the previous trial received 250 mmol of bicarbonate in the first 24 h [10]. In contrast, patients in the SB group of the present study received only 110 mmol.

The difference might be explained by the study design and the countries where the studies were conducted. The previous trial, mandated correction of pH, was an interventional study and was conducted in France. The present study was an observational study that described current clinical practice in a non-trial, real world setting and was conducted in Australia, Japan, and Taiwan. Sodium bicarbonate is provided in a 100-mL bottle in Australia, which might have led to a fixed dose treatment with 100 mL of sodium bicarbonate (= 100 mmol). In Japan and Taiwan, the BMIs of the general population are lower than that in the previous French trial (median BMI, 26). Clinicians in these countries might give a lower dose of sodium bicarbonate than in France by default, albeit they also do not appear to adjust the dose by the patient’s weight.

### Implications for clinicians

Our findings imply that early metabolic acidosis is relatively common in critically ill patients, as is early sodium bicarbonate therapy. However, they also imply that the speed and mode of administration of sodium bicarbonate are highly variable and that its dose is not adjusted for the degree of acidosis or the patient’s weight. The lack of a significant signal of harm in using sodium bicarbonate, the greater improvement in biochemical derangements and PaO2/FiO2 ratio, and the greater increase in MAP among vasopressor-dependent patients all imply that bicarbonate therapy in early metabolic acidosis may be safe. Finally, the aOR for ICU mortality among vasopressor-dependent patients favored sodium bicarbonate but with the wide confidence interval. This finding provides a rationale for further investigations of intravenous sodium bicarbonate therapy in vasopressor-treated patients with metabolic acidosis.

### Strengths and limitations

This is the first international multicenter epidemiological study to describe the incidence and clinical management of early metabolic acidosis with early sodium bicarbonate in the ICU. Despite the seeming frequency of such clinical practice, the effect of sodium bicarbonate on blood pressure and other biochemical parameters have not been assessed adequately [9]. This study, therefore, revealed a number of previously unknown aspects of both early metabolic acidosis and early sodium bicarbonate therapy that can be used to design double-blind, randomized controlled trials.

Several limitations should be acknowledged. First, due to the nature of the observational study design, the results of this analysis are prone to unmeasured confounding, and some assumptions required for the models used in the analysis. Second, we only treated early administration of sodium bicarbonate as being within 24 h of metabolic acidosis. This might increase the risk of failure to separate the two groups in assessing outcomes. Considering the evolution of metabolic acidosis in critical illness and the rapid/intense interventions provided in the ICU at the early phase of admission, we estimated 24 h would be sufficient for initial metabolic resuscitation. Furthermore, pH, BE, and HCO_3_ levels between the two groups at 24 h were similar, suggesting that later administration of sodium bicarbonate, even if given, would not have had a significant impact on the outcome. Third, creatinine levels were not measured at 24 h after the diagnosis of metabolic acidosis in many patients; thus, we could not assess the progression of AKI stage over this period.

Fourth, as this is not a randomized trial, any adjusted association observed cannot be taken to indicate causation. Thus, all findings from this study should be taken as exploratory and hypothesis-generating. Fifth, the sample size was not calculated due to the lack of epidemiological data on current clinical practice, so that limited data were available in patients with vasopressor dependency or severe metabolic acidosis at the diagnosis of metabolic acidosis. In reference to the subgroup of severe metabolic acidosis, only 13% of the overall study population was included. The findings in such a small group are not sufficiently robust to be informative. Finally, the findings from the subgroups should be interpreted cautiously, as multiple testing was performed in the study, and the level of statistical significance was not corrected for multiplicity [13].

## Conclusion

Early metabolic acidosis was common in ICU patients, and sodium bicarbonate was preferentially given to more severely ill patients. However, sodium bicarbonate therapy was variable in speed and mode of delivery and not adjusted for the patient’s body weight or the severity of metabolic acidosis. Despite such shortcomings in its dosage and delivery, sodium bicarbonate was independently associated with faster resolution of acid–base derangements and PaO2/FiO2 ratio. Moreover, in vasopressor-dependent patients, it was independently associated with a significantly greater increase in mean arterial pressure at six hours and a non-significant but favorable association with decreased ICU mortality. In their aggregate, these observations support the conduct of further controlled trials of bicarbonate therapy in early metabolic acidosis of critical illness.

## Supplementary information

**Additional file 1.** Supplementary materials include detailed methods and supplementary results as referenced inthe article.

## Data Availability

The datasets used and/or analyzed during the current study are available from the corresponding author on reasonable request.

## Abbreviations

RRT: renal replacement therapy
ICU: intensive care unit
BE: base excess
APACHE: acute physiology and chronic health evaluation
SB: Sodium bicarbonate
AKI: acute kidney injury
SID: strong ion difference
IQR: interquartile range
OR: odds ratio
