# Supplementary material for: Incidence and management of metabolic acidosis with sodium bicarbonate in the ICU: An international observational study
Source: Crit Care. 2021 Feb 2;25:45. doi: 10.1186/s13054-020-03431-2 (PMC7851901; doi:10.1186/s13054-020-03431-2)
Supplement: Supplementary file 1 — Additional file 1. Supplementary materials include detailed methods and supplementary results as referenced inthe article. [file 13054_2020_3431_MOESM1_ESM.pdf]

Supplement to: Fujii T, Udy AA, Nichol A, et al. Incidence and management of metabolic acidosis with sodium bicarbonate in the ICU: An international observational study.

**Supplementary Figure 1.** Diagram of screening and patient data collection. Every patient meeting the criteria were registered until 10 patients in each group (NaHCO3 vs no-NaHCO3)

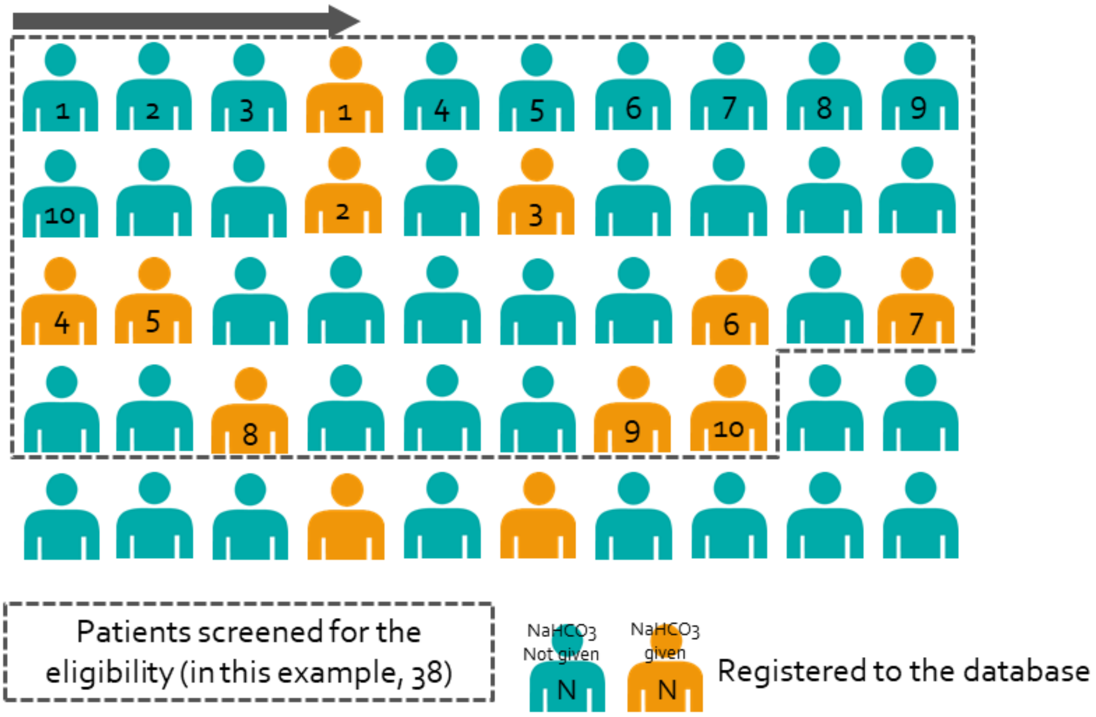

Supplement to: Fujii T, Udy AA, Nichol A, et al. Incidence and management of metabolic acidosis with sodium bicarbonate in the ICU: An international observational study.

**Supplementary Figure 2.** The total amount of sodium bicarbonate by body weight, base excess or bicarbonate levels.

**a. By body weight**

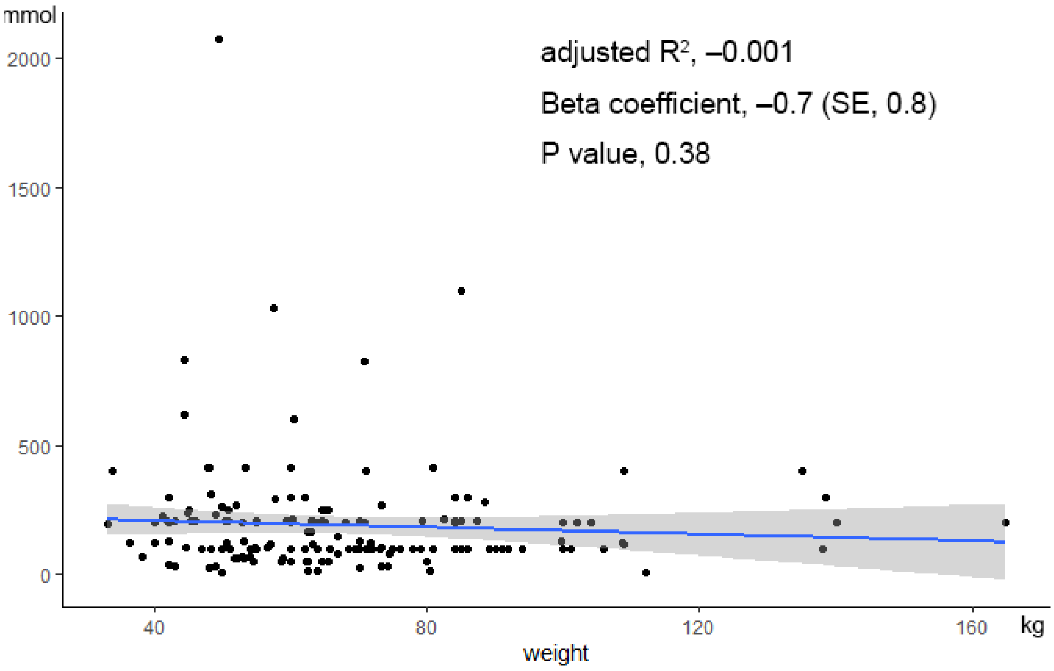


**b. By base excess**

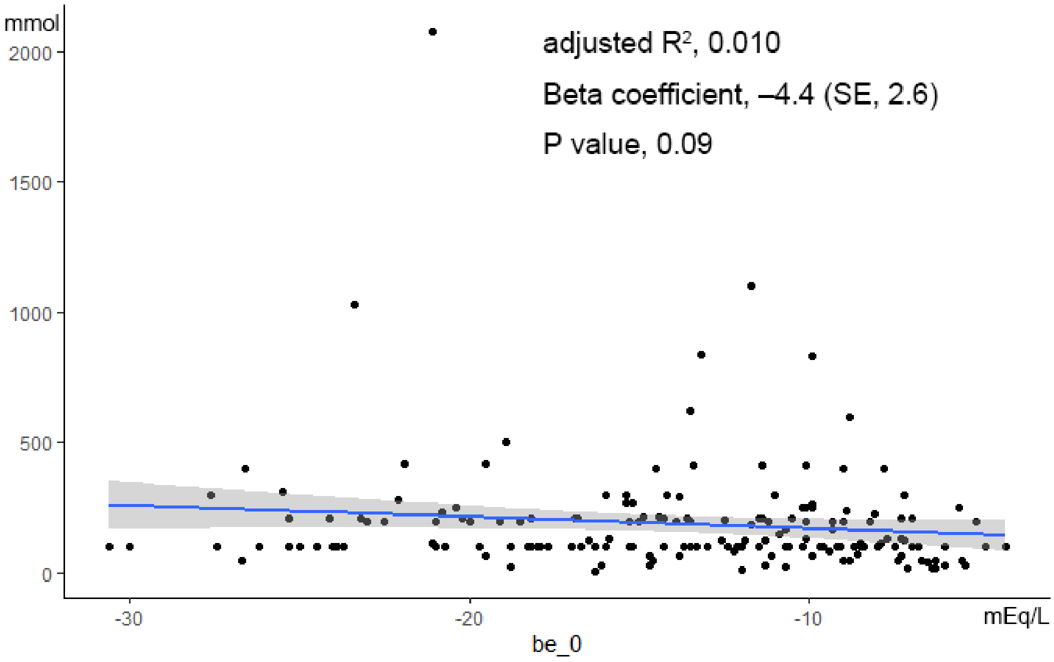

Supplement to: Fujii T, Udy AA, Nichol A, et al. Incidence and management of metabolic acidosis with sodium bicarbonate in the ICU: An international observational study.

**c. By bicarbonate levels**

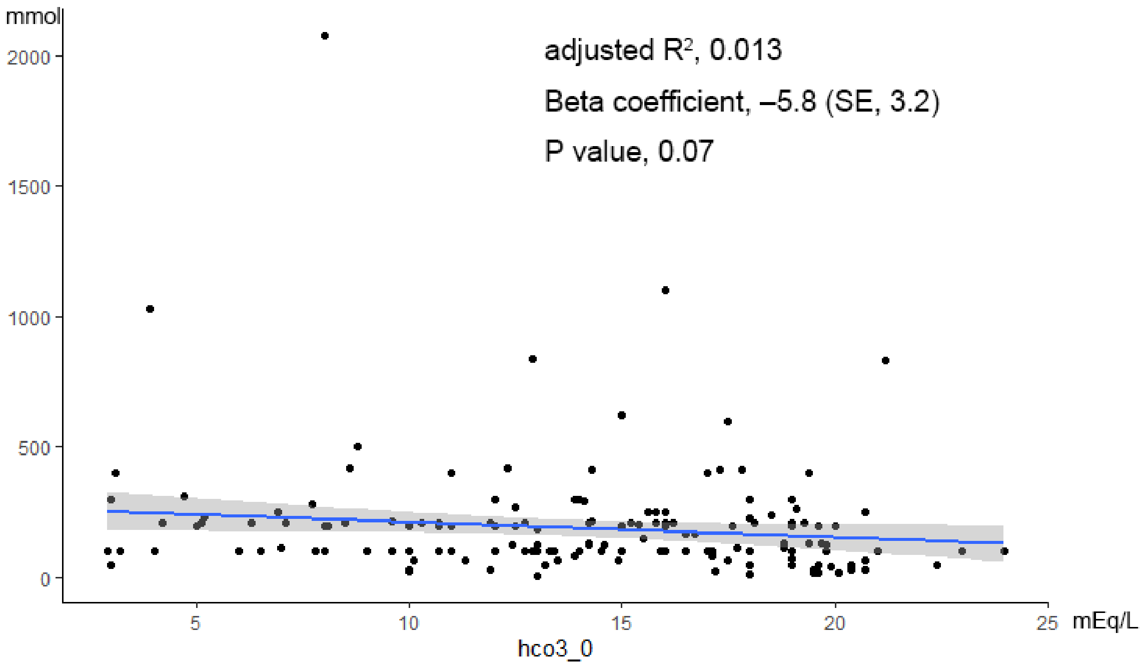

Supplement to: Fujii T, Udy AA, Nichol A, et al. Incidence and management of metabolic acidosis with sodium bicarbonate in the ICU: An international observational study.

**Supplementary Figure 3.** Need for renal replacement therapy over 24 hours of metabolic acidosis by treatment group.

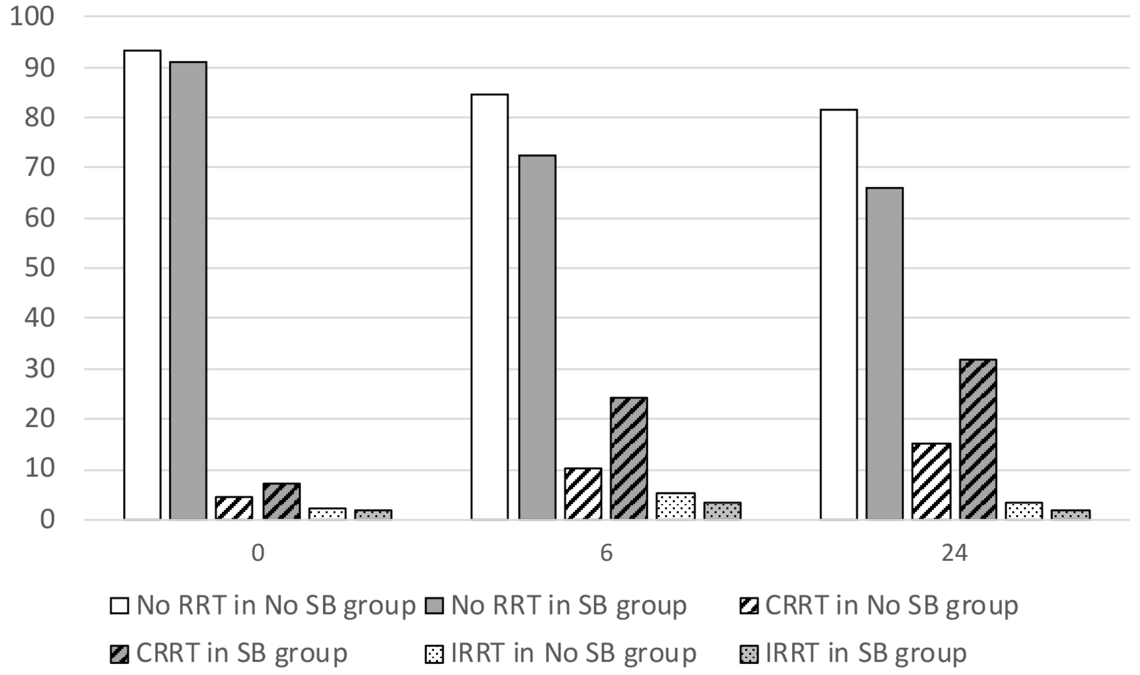

Supplement to: Fujii T, Udy AA, Nichol A, et al. Incidence and management of metabolic acidosis with sodium bicarbonate in the ICU: An international observational study.

**Supplementary Figure 4.** Distribution of patients with severe metabolic acidosis. Red bar = overall population, blue bar= subpopulation with the criteria.

a. pH < 7.2 and PaCO2 ≤ 45 mmHg

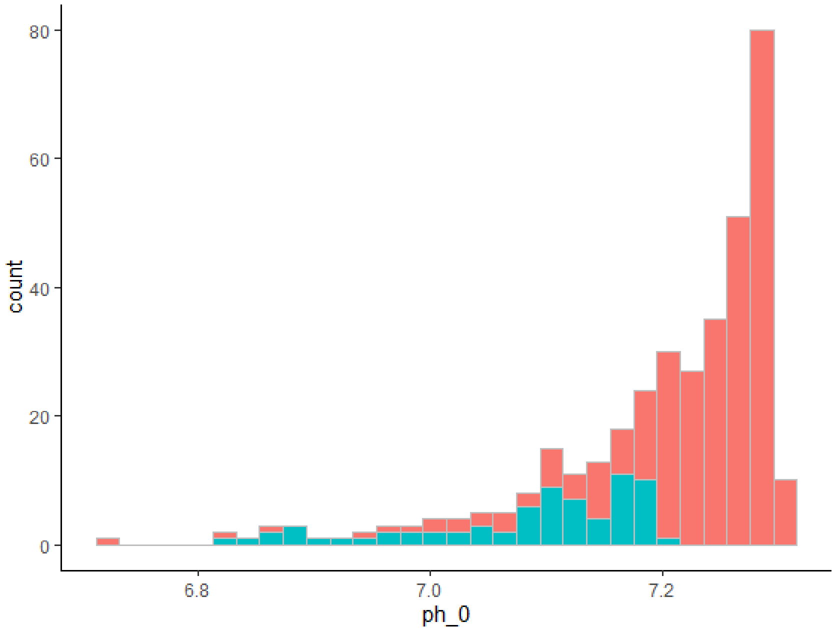


b. pH < 7.2AND PaCO2 ≤ 45 mmHg AND HCO3 ≤ 20mEq/L AND Lactate ≥ 2.0 mmol/L

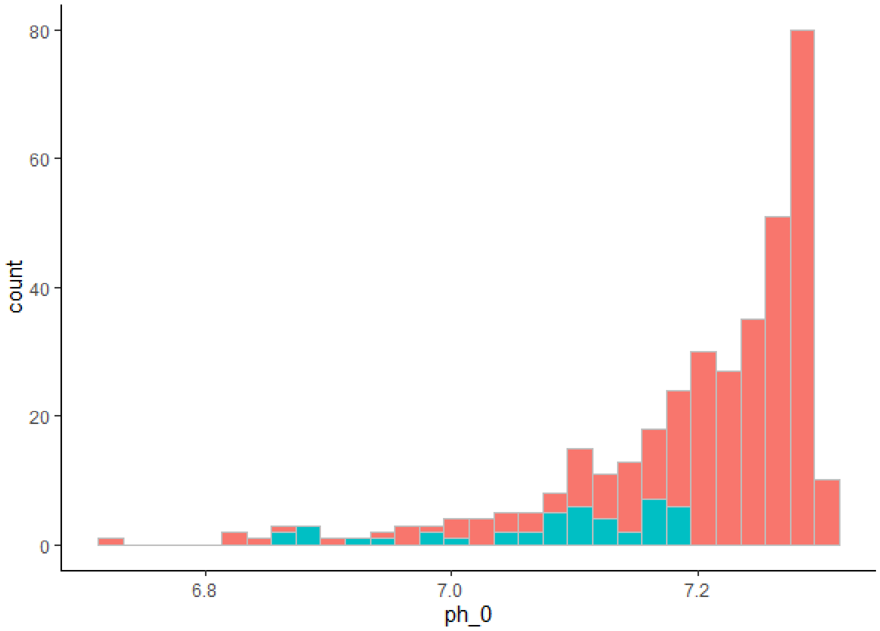

Supplement to: Fujii T, Udy AA, Nichol A, et al. Incidence and management of metabolic acidosis with sodium bicarbonate in the ICU: An international observational study.

**Supplementary Table 1.** Characteristics at ICU admission in patients with vasopressor dependency at the diagnosis of metabolic acidosis

| | No SB (N=77) | SB (N=110) | p value |
|---|---|---|---|
| Age, years | 69.6 [59.4, 81.0] | 66.1 [53.0, 76.1] | 0.04 |
| Sex, male | 52 (67.5) | 60 (54.5) | 0.10 |
| Weight, kg | 73.0 [59.2, 85.0] | 64.0 [52.0, 81.0] | 0.05 |
| Height, cm | 164 [158, 172] | 166 [155, 172] | 0.99 |
| ICU admission source | | | 0.05 |
| Operation theatre | 32 (41.6) | 24 (21.8) | |
| Emergency department | 22 (28.6) | 46 (41.8) | |
| Ward | 16 (20.8) | 32 (29.1) | |
| Other hospital | 5 ( 6.5) | 6 ( 5.5) | |
| ICU in other hospital | 2 ( 2.6) | 2 ( 1.8) | |
| Treatment goals on admission | | | 0.21 |
| Full active management | 68 (88.3) | 94 (85.5) | |
| Treatment limitation order | 9 (11.7) | 10 ( 9.1) | |
| Palliative care | 0 ( 0.0) | 5 ( 4.5) | |
| Potential organ donation | 0 ( 0.0) | 1 ( 0.9) | |
| Cardiac arrest in 24 hours prior to ICU admission | | | 0.34 |
| Yes | 14 (18.2) | 28 (25.5) | |
| No | 63 (81.8) | 81 (73.6) | |
| Unknown | 0 ( 0.0) | 1 ( 0.9) | |
| Chronic hemo/peritoneal-dialysis | 6 ( 7.8) | 4 ( 3.6) | 0.36 |
| Baseline creatinine, mmol/L | 93.0 [74.3, 185.4] | 97.0 [57.1, 142.0] | 0.27 |
| AKI | | | 0.08 |
| No AKI | 24 (45.3) | 23 (29.5) | |
| Stage 1 | 15 (28.3) | 17 (21.8) | |
| Stage 2 | 3 ( 5.7) | 8 (10.3) | |
| Stage 3 | 11 (20.8) | 30 (38.5) | |
| APACHE III score | 70 [51, 95] | 99 [71, 129] | <0.001 |

**Supplementary Table 2.** Arterial blood gas analysis and electrolytes in patients with vasopressor dependency at the diagnosis of metabolic acidosis. pH, PaO2, PaCO2, HCO3, Base Excess, and lactate were only measured for arterial blood samples.

| | No SB (N=77) | SB (N=110) | p value |
|---|---|---|---|
| pH | 7.26 [7.22, 7.28] | 7.19 [7.13, 7.25] | <0.001 |
| PaO2, mmHg | 109.7 [83.1, 182.2] | 113.8 [82.4, 209.8] | 0.45 |
| PaO2/FiO2 | 238.8 [144.8, 323.3] | 230.9 [126.8, 370.8] | 0.95 |
| PaCO2, mmHg | 43.8 [37.6, 49.0] | 39.5 [32.1, 47.3] | 0.04 |
| HCO3, mEq/L | 18.2 [16.0, 20.7] | 15.0 [11.9, 18.0] | <0.001 |
| Base Excess, mEq/L | -7.9 [-10.0, -6.0] | -12.3 [-16.1, -9.3] | <0.001 |
| SID, mEq/L | 29.7 [25.8, 32.5] | 27.6 [23.4, 31.6] | 0.03 |
| Sodium, mEq/L | 137.0 [135.0, 140.0] | 137.0 [134.0, 140.0] | 0.59 |
| Potassium, mEq/L | 4.2 [3.8, 4.8] | 4.3 [3.8, 5.1] | 0.67 |
| Chloride, mEq/L | 109.0 [106.3, 113.0] | 107.0 [103.0, 112.0] | 0.07 |
| Lactate, mmol/L | 2.9 [1.8, 5.1] | 6.6 [2.3, 10.2] | 0.001 |
| Ionised Calcium, mmol/L | 1.1 [1.0, 1.2] | 1.1 [1.0, 1.2] | 0.62 |

SB denotes sodium bicarbonate; SID, strong ion difference = Sodium + Potassium + Ionised calcium - Chloride – Lactate.

**Supplementary Table 3.** ICU interventions in patients with vasopressor dependency at the time of metabolic acidosis diagnosis

| | No SB (N=77) | SB (N=110) | p value |
|---|---|---|---|
| Vasopressor dose, µg/kg/min | 0.12 [0.05, 0.28] | 0.25 [0.13, 0.47] | <0.001 |
| Cardiac index monitored | 8 (12.1) | 11 (12.2) | > 0.99 |
| Cardiac index, L/min/$m^2$ | 2.6 [2.4, 3.0] | 3.2 [2.4, 3.8] | 0.33 |
| Invasive mechanical ventilation | 60 (77.9) | 82 (74.5) | |
| Noninvasive mechanical ventilation | 3 ( 3.9) | 1 ( 0.9) | |
| ECMO | 0 ( 0.0) | 5 ( 4.5) | |
| Neuromuscular blockade | 13 (17.1) | 12 (11.0) | 0.33 |
| Renal replacement therapy | | | 0.71 |
| Not on RRT | 69 (90.8) | 96 (87.3) | |
| CRRT | 6 ( 7.9) | 11 (10.0) | |
| IRRT | 1 ( 1.3) | 3 ( 2.7) | |

SB denotes sodium bicarbonate; ECMO, extracorporeal membrane oxygenation; RRT, renal replacement therapy; CRRT, continuous renal replacement therapy; IRRT, intermittent renal replacement therapy.

Supplement to: Fujii T, Udy AA, Nichol A, et al. Incidence and management of metabolic acidosis with sodium bicarbonate in the ICU: An international observational study.

**Supplementary Table 4.** Characteristics at ICU admission in patients with severe metabolic acidosis.

| | No SB (N=7) | SB (N=39) | p value |
|---|---|---|---|
| Age, years | 52.4 [47.7, 65.2] | 63.3 [49.2, 70.8] | 0.47 |
| Sex, male | 3 (42.9) | 13 (33.3) | 0.68 |
| Weight, kg | 74.0 [63.5, 81.2] | 63.3 [53.8, 80.9] | 0.22 |
| Height, cm | 162 [160, 172] | 162 [156, 170] | 0.76 |
| BMI, kg/m$^2$ | 25.0 [25.0, 28.1] | 22.3 [21.1, 26.6] | 0.09 |
| ICU admission source | | | 0.80 |
| Operation theatre | 1 (14.3) | 3 (7.7) | |
| Emergency department | 4 (57.1) | 24 (61.5) | |
| Ward | 2 (28.6) | 7 (17.9) | |
| Other hospital | 0 (0) | 2 (5.1) | |
| ICU in other hospital | 0 (0) | 3 (7.7) | |
| Treatment goals on admission | | | >0.999 |
| Full active management | 7 (100) | 32 (82.1) | |
| Treatment limitation order | 0 (0) | 4 (10.3) | |
| Palliative care | 0 (0) | 3 (7.7) | |
| Cardiac arrest in 24 hours prior to ICU admission | 3 (42.9) | 10 (25.6) | 0.39 |
| Chronic hemo/peritoneal-dialysis | 4 (57.1) | 3 (7.7) | 0.01 |
| Baseline creatinine, mmol/L | 259.0 [104.0, 342.0] | 117.5 [72.6, 157.4] | 0.23 |
| AKI | | | 0.70 |
| No AKI | 3 (60.0) | 8 (29.6) | |
| Stage 1 | 0 (0) | 5 (18.5) | |
| Stage 2 | 0 (0) | 5 (18.5) | |
| Stage 3 | 2 (40.0) | 9 (33.3) | |
| APACHE III score | 96 [81, 120] | 106 [92, 134] | 0.56 |

**Supplementary Table 5.** Arterial blood gas analysis and electrolytes in patients with severe metabolic acidosis. pH, PaO2, PaCO2, HCO3, Base Excess, and lactate were only measured for arterial blood samples.

| | No SB (N=7) | SB (N=39) | p value |
|---|---|---|---|
| pH | 7.12 [7.04, 7.19] | 7.11 [7.06, 7.16] | 0.42 |
| PaO2, mmHg | 87.5 [83.7, 257.7] | 153.0 [113.1, 288.0] | 0.42 |
| PaO2/FiO2 | 142.2 [136.7, 284.9] | 310.0 [215.3, 456.9] | 0.12 |
| PaCO2, mmHg | 36.6 [27.8, 38.0] | 33.0 [26.0, 39.2] | 0.80 |
| HCO3, mEq/L | 13.0 [6.6, 13.8] | 9.6 [6.7, 12.5] | 0.49 |
| Base Excess, mEq/L | –15.2 [–24.5, –13.6] | –19.5 [–23.7, –16.3] | 0.58 |
| SID, mEq/L | 25.4 [21.4, 27.6] | 24.1 [21.0, 29.1] | 0.80 |
| Sodium, mEq/L | 134.0 [126.8, 136.5] | 136.0 [133.5, 138.0] | 0.15 |
| Potassium, mEq/L | 5.2 [4.1, 6.0] | 4.5 [4.1, 4.9] | 0.33 |
| Chloride, mEq/L | 99.0 [96.0, 106.5] | 105.0 [100.0, 111.5] | 0.13 |
| Lactate, mmol/L | 7.0 [4.2, 16.6] | 9.0 [4.5, 13.4] | 0.95 |
| Ionised Calcium, mmol/L | 1.1 [1.1, 1.2] | 1.1 [1.0, 1.2] | 0.13 |

SB denotes sodium bicarbonate; SID, strong ion difference = Sodium + Potassium + Ionised calcium - Chloride – Lactate.

**Supplementary Table 6.** ICU interventions in patients with severe metabolic acidosis at the time of metabolic acidosis diagnosis

| | No SB (N=7) | SB (N=39) | p value |
|---|---|---|---|
| Vasopressor dose, µg/kg/min | 0.00 [0.00, 0.22] | 0.11 [0.00, 0.40] | 0.28 |
| Cardiac index monitored | 0 (0) | 0 (0) | N/A |
| Respiratory support | | | 0.51 |
| Not on mechanical ventilation | 2 (28.6) | 12 (30.8) | |
| Invasive mechanical ventilation | 4 (57.1) | 25 (64.1) | |
| Noninvasive mechanical ventilation | 1 (14.3) | 1 (2.6) | |
| ECMO | 0 (0) | 1 (2.6) | |
| Neuromuscular blockade | 2 (28.6) | 3 (7.7) | 0.16 |
| Renal replacement therapy | | | 0.57 |
| Not on RRT | 7 (100) | 33 (84.6) | |
| CRRT | 0 (0) | 6 (15.4) | |

SB denotes sodium bicarbonate; N/A, not applicable; ECMO, extracorporeal membrane oxygenation; RRT, renal replacement therapy; CRRT, continuous renal replacement therapy.

**Supplementary Table 7.** Characteristics at ICU admission in patients with Stage 2 or 3 AKI at the diagnosis of metabolic acidosis

| | No SB (N=34) | SB (N=50) | p value |
|---|---|---|---|
| Age, years | 67.6 [55.3, 80.3] | 67.3 [55.4, 72.7] | 0.43 |
| Sex, male | 26 ( 76.5) | 23 ( 46.0) | 0.01 |
| Weight, kg | 61.8 [54.4, 76.4] | 63.6 [53.1, 74.0] | 0.91 |
| Height, cm | 166.5 [156.6, 174.3] | 164.0 [149.6, 171.5] | 0.17 |
| ICU admission source | | | 0.92 |
| Operation theatre | 8 ( 23.5) | 9 ( 18.0) | |
| Emergency department | 14 ( 41.2) | 19 ( 38.0) | |
| Ward | 10 ( 29.4) | 19 ( 38.0) | |
| Other hospital | 1 ( 2.9) | 1 ( 2.0) | |
| ICU in other hospital | 1 ( 2.9) | 2 ( 4.0) | |
| Treatment goals on admission | | | 0.46 |
| Full active management | 28 ( 82.4) | 41 ( 82.0) | |
| Treatment limitation order | 6 ( 17.6) | 7 ( 14.0) | |
| Palliative care | 0 ( 0.0) | 2 ( 4.0) | |
| Cardiac arrest in 24 hours prior to ICU admission | 5 ( 14.7) | 8 ( 16.0) | >0.99 |
| Chronic hemo/peritoneal-dialysis | 13 ( 39.4) | 3 ( 6.0) | <0.001 |
| Baseline creatinine, mmol/L | 271.4 [98.7, 524.9] | 87.2 [54.2, 144.3] | <0.001 |
| AKI | | | >0.99 |
| Stage 2 | 8 ( 23.5) | 11 (22.0) | |
| Stage 3 | 26 ( 76.5) | 39 ( 78.0) | |
| APACHE III score | 91.5 [69.3, 107.3] | 110.0 [88.0, 144.5] | 0.002 |

**Supplementary Table 8.** Arterial blood gas analysis and electrolytes in patients with Stage 2 or 3 AKI. pH, PaO2, PaCO2, HCO3, Base Excess, and lactate were only measured for arterial blood samples.

| | No SB (N=34) | SB (N=50) | p value |
|---|---|---|---|
| pH | 7.27 [7.20, 7.29] | 7.19 [7.12, 7.25] | 0.001 |
| PaO2, mmHg | 111.50 [82.83, 143.22] | 109.90 [83.10, 158.25] | 0.902 |
| PaO2/FiO2 | 251.23 [182.46, 342.11] | 255.00 [150.00, 372.22] | 0.86 |
| PaCO2, mmHg | 38.05 [35.80, 46.38] | 37.05 [31.05, 43.38] | 0.314 |
| HCO3, mEq/L | 16.90 [14.40, 20.48] | 13.95 [10.03, 17.18] | 0.001 |
| Base Excess, mEq/L | -8.95 [-12.72, -6.27] | -13.45 [-17.88, -10.05] | <0.001 |
| SID, mEq/L | 32.89 [28.02, 36.30] | 27.95 [23.57, 31.85] | 0.066 |
| Sodium, mEq/L | 137.00 [134.00, 140.00] | 136.00 [133.00, 138.00] | 0.22 |
| Potassium, mEq/L | 4.70 [4.16, 5.43] | 4.30 [3.80, 5.12] | 0.108 |
| Chloride, mEq/L | 107.00 [104.50, 112.50] | 104.00 [102.00, 110.00] | 0.103 |
| Lactate, mmol/L | 1.60 [1.20, 4.05] | 6.33 [1.40, 11.38] | 0.022 |
| Ionised Calcium, mmol/L | 1.12 [1.06, 1.21] | 1.11 [1.02, 1.21] | 0.271 |

SB denotes sodium bicarbonate; SID, strong ion difference = Sodium + Potassium + Ionised calcium - Chloride – Lactate.

**Supplementary Table 9.** ICU interventions in patients with Stage 2 or 3 AKI at the time of metabolic acidosis diagnosis

| | No SB (N=34) | SB (N=50) | p value |
|---|---|---|---|
| Vasopressor dose, μg/kg/min | 0.00 [0.00, 0.10] | 0.20 [0.01, 0.35] | 0.001 |
| Cardiac index monitored | 0 ( 0.0) | 6 ( 12.0) | 0.10 |
| Respiratory support | | | 0.61 |
| Not on mechanical ventilation | 17 ( 50.0) | 20 ( 40.0) | |
| Invasive mechanical ventilation | 16 ( 47.1) | 29 ( 58.0) | |
| Noninvasive mechanical ventilation | 1 ( 2.9) | 1 ( 2.0) | |
| Neuromuscular blockade | 6 ( 17.6) | 3 ( 6.1) | 0.19 |
| Renal replacement therapy | | | 0.57 |
| Not on RRT | 22 ( 64.7) | 34 ( 68.0) | |
| CRRT | 8 ( 23.5) | 13 ( 26.0) | |
| IRRT | 4 ( 11.8) | 3 ( 6.0) | |

SB denotes sodium bicarbonate; N/A, not applicable; ECMO, extracorporeal membrane oxygenation; RRT, renal replacement therapy; CRRT, continuous renal replacement therapy; IRRT, intermittent renal replacement therapy.

**Supplementary Table 10**. Association between administration of sodium bicarbonate and clinical outcomes in patients with Stage 2 or 3 AKI (N = 84).

| | aOR, β | 95% CI | p-value |
|---|---|---|---|
| ICU mortality | 0.72 | 0.21 to 2.58 | 0.59 |
| Hospital mortality | 1.13 | 0.37 to 4.14 | 0.83 |
| Vasopressor dose at 6h | 0.06 | –0.02 to 0.14 | 0.15 |
| Vasopressor dose at 24h | 0.07 | –0.02 to 0.16 | 0.15 |
| Mean arterial pressure at 6h | –1.13 | –8.09 to 5.39 | 0.73 |
| Mean arterial pressure at 24h | –6.54 | –13.49 to 0.42 | 0.07 |
| Delta mean arterial pressure per vasopressor dose at 6h | 1.89 | –6.85 to 10.41 | 0.67 |
| Delta mean arterial pressure per vasopressor dose at 24h | 2.83 | –7.53 to 13.19 | 0.60 |

Delta mean arterial pressure per vasopressor dose = mean arterial pressure at 24h/(vasopressor dose at 24h +1) – mean arterial pressure at 6h/(vasopressor dose at 6h +1)
